# CENTERING DIVERSE AGING ADULTS: STRENGTH, RESILIENCE, AND OPPORTUNITIES

**DOI:** 10.1093/geroni/igae098.0885

**Published:** 2024-12-31

**Authors:** Elizabeth Muñoz, Amy Thierry

**Affiliations:** Chair; Co-Chair

## Abstract

Our older adult population is increasingly becoming more diverse and there is a need to identify sources of risk and resilience among individuals living at the intersection of multiple systems of oppression or adversity. This symposium highlights the lived experiences of older diverse adults through a multi-dimensional lens by investigating the experiences of adults across community settings, exposure to trauma, identity (gender and race), and health status. Presentations also investigate already-existing strengths within these communities and recommend opportunities for leveraging those assets to bolster health equity in diverse older adults. To this end, Tian and colleagues will present results on coping strategy utilization and stress among urban-dwelling diverse adults during the COVID-19 pandemic. Cherry and colleagues will showcase an investigation of the experiences of African American / Black women living in Louisiana who endured the 2005 hurricanes and the COVID-19 pandemic. Wright and colleagues will share findings on the association between parental education, adverse childhood experiences, and memory performance among diverse, community-dwelling older adults. Groves and colleagues will present findings on the feasibility of a dyadic intervention to support hypertension self-management among older African American women with hypertension. Lastly, Gaillard and colleagues identify strategies for increasing engagement in health research among older African Americans. Overall, this symposium centers the lived experiences, strengths, and resilience of diverse older adults, and points to novel directions for advancing health equity research.

